# Supplementary material for: Empirical methods for controlling false positives and estimating confidence in ChIP-Seq peaks
Source: BMC Bioinformatics. 2008 Dec 5;9:523. doi: 10.1186/1471-2105-9-523 (PMC2628906; doi:10.1186/1471-2105-9-523)
Supplement: Additional File 1 — A variety of html documents from the USeq web site detailing the available applications, their best usage, output file type descriptions, command line menus, etc. [file 1471-2105-9-523-S1.zip › USeqUserGuides/JavaDoc.zip!/JavaDoc/trans/tpmap/OligoFlipper.html]

OligoFlipper


|  |  |  |
| --- | --- | --- |
| **Overview** \| **Package** \| **Class** \| **Use** \| **Tree** \| **Deprecated** \| **Index** \| **Help** | |  |
| **PREV CLASS**   **NEXT CLASS** | **FRAMES**    **NO FRAMES**     **All Classes** |  |
| SUMMARY: NESTED \| FIELD \| CONSTR \| METHOD | DETAIL: FIELD \| CONSTR \| METHOD |  |


---


## trans.tpmap Class OligoFlipper

```
java.lang.Object
  trans.tpmap.OligoFlipper
```

---

``` public class OligoFlipper extends java.lang.Object ```

This reversed the oligo sequence and orientation in a tpmap file.

---

**Constructor Summary**

`OligoFlipper(java.lang.String[] args)`


| **Method Summary** | |
| --- | --- |
| `static void` | `main(java.lang.String[] args)` |
| `static void` | `printDocs()` |
| `void` | `processArgs(java.lang.String[] args)`             This method will process each argument and assign any new varibles |

**Methods inherited from class java.lang.Object**

`equals, getClass, hashCode, notify, notifyAll, toString, wait, wait, wait`

**Constructor Detail**

### OligoFlipper

```
public OligoFlipper(java.lang.String[] args)
```


**Method Detail**

### processArgs

```
public void processArgs(java.lang.String[] args)
```

:   This method will process each argument and assign any new varibles

---


### printDocs

```
public static void printDocs()
```

---


### main

```
public static void main(java.lang.String[] args)
```


---


|  |  |  |
| --- | --- | --- |
| **Overview** \| **Package** \| **Class** \| **Use** \| **Tree** \| **Deprecated** \| **Index** \| **Help** | |  |
| **PREV CLASS**   **NEXT CLASS** | **FRAMES**    **NO FRAMES**     **All Classes** |  |
| SUMMARY: NESTED \| FIELD \| CONSTR \| METHOD | DETAIL: FIELD \| CONSTR \| METHOD |  |


---
